# Association of rs610604 in TNFAIP3 and rs17728338 in TNIP1 gene polymorphisms with psoriasis susceptibility: a meta-analysis of case-control studies

**DOI:** 10.1186/s12881-020-01041-x

**Published:** 2020-05-12

**Authors:** Hai-bo Gong, Shu-tao Gao, Xiong-ming Pu, Xiao-jing Kang, Xiu-juan Wu

**Affiliations:** 1 Department of Dermatology, People’s Hospital of Xinjiang Uygur Autonomous Region, 91 Tianchi Road, Tianshan District, Urumqi, 830001 Xinjiang China; 2 Department of Spine Surgery, The First Affiliated Hospital of Xinjiang Medical University, Urumqi, 830054 Xinjiang China; 3 Department of Dermatology, Shanghai Xuhui Central Hospital, Shanghai, 200031 China

**Keywords:** Psoriasis, TNFAIP3, TNIP1 single nucleotide polymorphism, Meta-analysis

## Abstract

**Background:**

To date, the fundamental pathophysiology underlying the occurrence and progression of psoriasis are still unanswered questions. Genome-wide association surveys have revealed that TNFAIP3 and TNIP1 were key biomarkers for psoriasis. Here, we intended to conduct a survey on the association between TNFAIP3 and TNIP1 gene polymorphisms and psoriasis risk.

**Methods:**

A comprehensive search of four online databases—China National Knowledge Infrastructure (CNKI), PubMed, Embase, and Cochrane Library was undertaken up to August 25, 2019. We chose allele genetic model to deal with the original data. Newcastle–Ottawa scale (NOS) was used to evaluate the risk bias of each study. The RevMan 5.3 software was used to calculate the combined odds ratio and 95% confidence interval.

**Results:**

In total, we included 13 case-control studies consist of 13,908 psoriasis patients and 20,051 controls in this work. Our results demonstrated that rs610604 in TNFAIP3 polymorphism was significantly associated with psoriasis risk using random-effect model (G vs. T, OR = 1.19, 95% CI: 1.09–1.31, *P* = 0.0002), and a significant association between rs17728338 in TNIP1 polymorphism and psoriasis vulnerability using fixed-effect model (A vs. G, OR = 1.69, 95% CI:1.58–1.80, *P* < 0.00001).

**Conclusions:**

Our findings indicated that rs610604 in TNFAIP3 and rs17728338 in TNIP1 gene polymorphisms were associated with psoriasis susceptibility.

## Background

Psoriasis is currently regarded as a chronic, inflammatory skin disease associated with systemic conditions [1–3]. As with other dermatoses, the patients who suffered from it also have to face the enormous psychological burden because of visible disfiguration. Considerable comorbid diseases often occur in psoriasis patients, including psoriatic arthritis, metabolic syndrome, cardiovascular disorders, gastrointestinal diseases, mood disorders as well as other emerging comorbid diseases [4]. Psoriasis affects approximately 2–3% of the population worldwide, and its prevalence is much higher in western countries [5, 6]. To date, five types of psoriasis have been identified: psoriasis vulgaris, guttate or eruptive psoriasis, inverse psoriasis, erythrodermic psoriasis and pustular psoriasis [3].

As a complex inflammatory disorder, the aetiology and pathogenesis of psoriasis are widely thought to be caused by the interplay of intrinsic and environmental factors. Numerous triggers and aggravations for psoriasis occurrence have been identified such as mild localized trauma, drugs, HIV infection and streptococcal pharyngitis [7]. However, intrinsic factors such as genetics may play a more important role. Thanks to the powerful genome-wide association studies (GWAS) as well as other genetic studies, more than 60 regions of the human genome have now been identified to be correlated with psoriasis [8]. Tumour Necrosis Factor Alpha-Induced Protein 3 (TNFAIP3) and TNFAIP3 Interacting Protein 1 (TNIP1) are among them, and they were first discovered to be associated with psoriasis in 2009 [9]. After that, numerous studies on the association of single nucleotide polymorphisms in TNFAIP3 and TNIP1 with the risk of psoriasis have conducted. However, the conclusions of these studies may be incomprehensive and contradictory. Thus, we aimed was to undertake a meta-analysis to analyze these studies comprehensively.

**Fig. 1:**
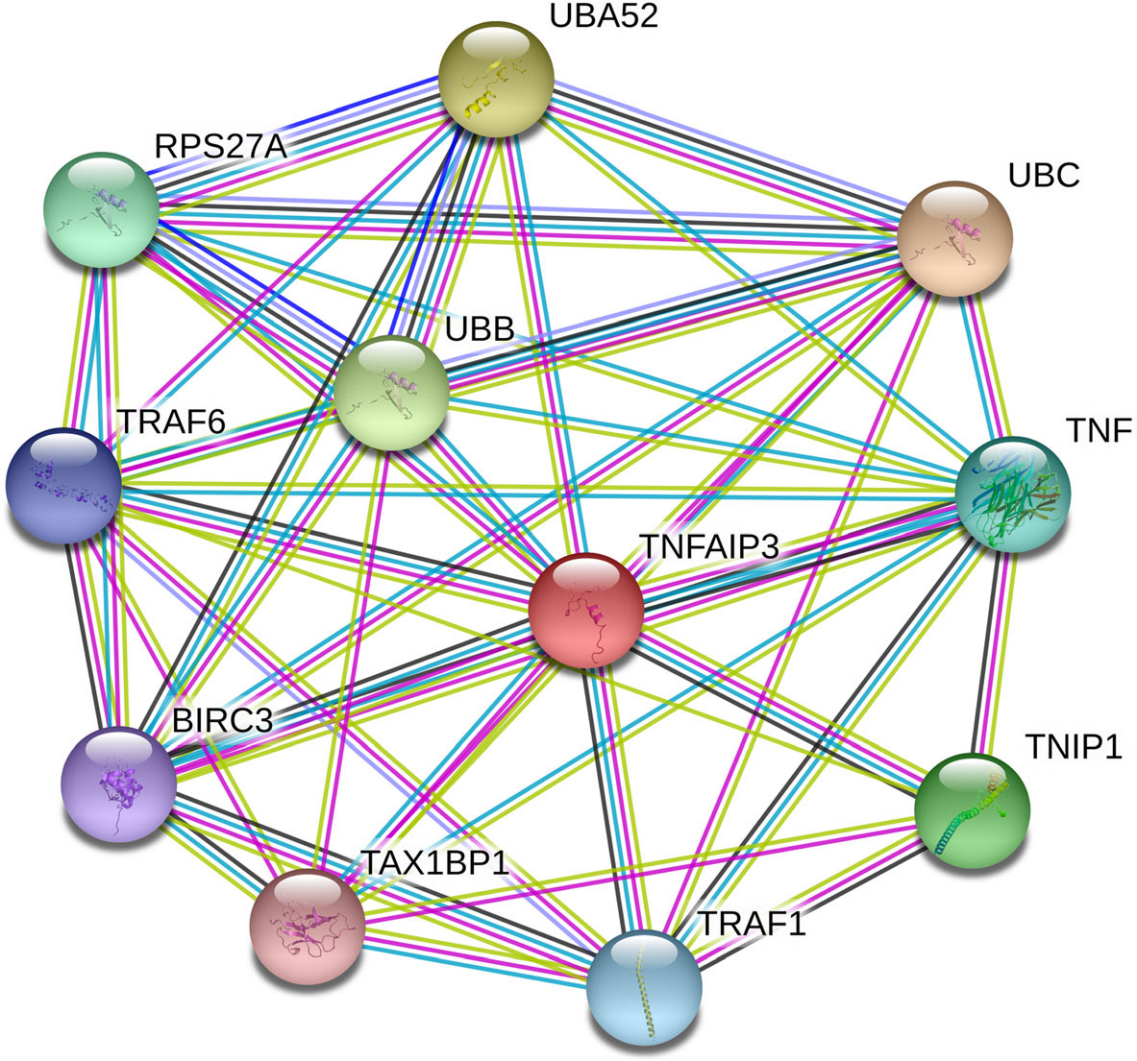
Network of TNFAIP3, TNIP1 and their closest functional partners. These data were from the Search Tool for the Retrieval of Interacting Genes (STRING) database (http://string-db.org/)

TNFAIP3 gene is located on human chromosome 6q23.3, another alias for TNFAIP3 is A20. While TNIP1 is located on 5q33.1. They are all Protein-Coding genes encode ubiquitin-editing enzyme A20 and A20-Binding Inhibitor Of NF-Kappa-B Activation 1 (ABIN-1), which interact with each other to influence intracellular signaling [10, 11]. Polymorphisms of the two genes may alter their protein-coding, and thus to have an impact on their closest functional protein partners. The interrelation network of TNFAIP3 and TNIP1 with their nearest associated functional protein partners were illustrated on Fig. 1. Over the years, accumulating evidence indicated that genetic variations in the genes TNFAIP3 and TNIP1 are strongly associated with vulnerability to numerous inflammatory diseases [12–14]. Considering that study sample sizes were small and the statistical effect was limited of an individual study, this meta-analysis is meant to provide the most comprehensive and precise evaluation on the association of TNFAIP3 and TNIP1 polymorphisms with psoriasis vulnerability.

## Methods

### Search strategy

Two of our investigators (Hai-bo Gong and Shu-tao Gao) independently did the literature searching among four major databases—CNKI, PubMed, Embase, and Cochrane Library—for papers published before August 25, 2019. The retrieval strategy of PubMed was as follows: ((((Psoriasis) OR “Psoriasis”[Mesh])) AND (((((((“Tumor Necrosis Factor alpha-Induced Protein 3”[Mesh]) OR Tumor Necrosis Factor alpha-Induced Protein 3) OR Zinc Finger Protein A20) OR A20) OR TNF Alpha-Induced Protein 3)) OR (((((((“TNIP1 protein, human” [Supplementary Concept]) OR TNIP1) OR TNFAIP3 Interacting Protein 1) OR A20-Binding Inhibitor Of NF-Kappa-B Activation 1) OR VAN protein, human) OR TNFalpha-induced protein 3-interacting protein 1, human) OR ABIN-1 protein, human))) AND ((((((single nucleotide polymorphism) OR Polymorphism) OR Alleles) OR SNP) OR Variation) OR gene).

### Inclusion and exclusion criteria

The main contents of our inclusion criteria are given as follows: published case-control studies on humans; psoriasis should be diagnosed according to clinical diagnosis criteria; studies related to the association of TNFAIP3 or TNIP1 polymorphisms with psoriasis susceptibility; contained sufficient original data to estimate odds ratios (ORs) and 95% confidence intervals (95% CIs). Accordingly, Studies met the following criteria should be excluded: reviews, conference abstracts, case reports; the exact number of alleles wasn’t able to be ascertained, and duplicated articles.

### Data extraction

The following information was extracted from each candidate study by two independent investigators (Hai-bo Gong and Shu-tao Gao) including: author’s names, year of publication, Ethnicity of the study population, numbers of cases and controls, and the allele frequencies of the TNFAIP3 or TNAP1 polymorphisms, Hardy-Weinberg equilibrium (HWE) results.

### Quality assessment

The methodological quality of each eligible study was evaluated by two investigators (Hai-bo Gong and Shu-tao Gao). Newcastle–Ottawa Scale (NOS) was used to appraise all included studies in line with its criteria. We used “Score system” to judge each study mainly made up of three aspects: selection (four items), comparability (two items), and the outcomes of case control studies (three items). Every included study received a total of 0–9 scores according to these items. A higher score indicated better quality. Studies with ≥5 scores were considered to have high quality for further analysis. When disagreements occurred between the two investigators, the third reviewer (Xiong-ming Pu) will be invited to make the final decision.

### Statistical analysis

The preferred Reporting Items for Systematic Review and Meta-analyses (PRISMA) was used to complete this meta-analysis [15]. We processed the initial data using allele model of inheritance due to a lack of sufficient information. The association between rs610604 in TNFAIP3 and rs17728338 in TNIP1 polymorphisms with psoriasis was estimated by pooled ORs with 95% CIs. Q-statistical test and *I*^2^ test were used to evaluate the heterogeneity among all included studies [16]. The random-effect model was used to combine the data in the cases of heterogeneity (*P* < 0.1, *I*^2^ > 50%) or fixed-effect model was used when it was out of heterogeneity (*P* >  0.1, *I*^2^ < 50%) [17, 18]. The Hardy–Weinberg Equilibrium results were either extracted from original studies or calculated by initial data in included studies. Sensitivity analysis was conducted using Stata 12.0 software (Stata Corp LP, U.S.A). Revman 5.3 software was used to generate Forest plots. Egger’s test was used to evaluate the publication bias by Stata version 12.0.

### Functional predictions

To dig deeper into the potentially functional role of these loci, we used in silico tool HaploReg 4.1 to explore the annotations in the human genome.

## Results

### Study characteristics

**Fig. 2:**
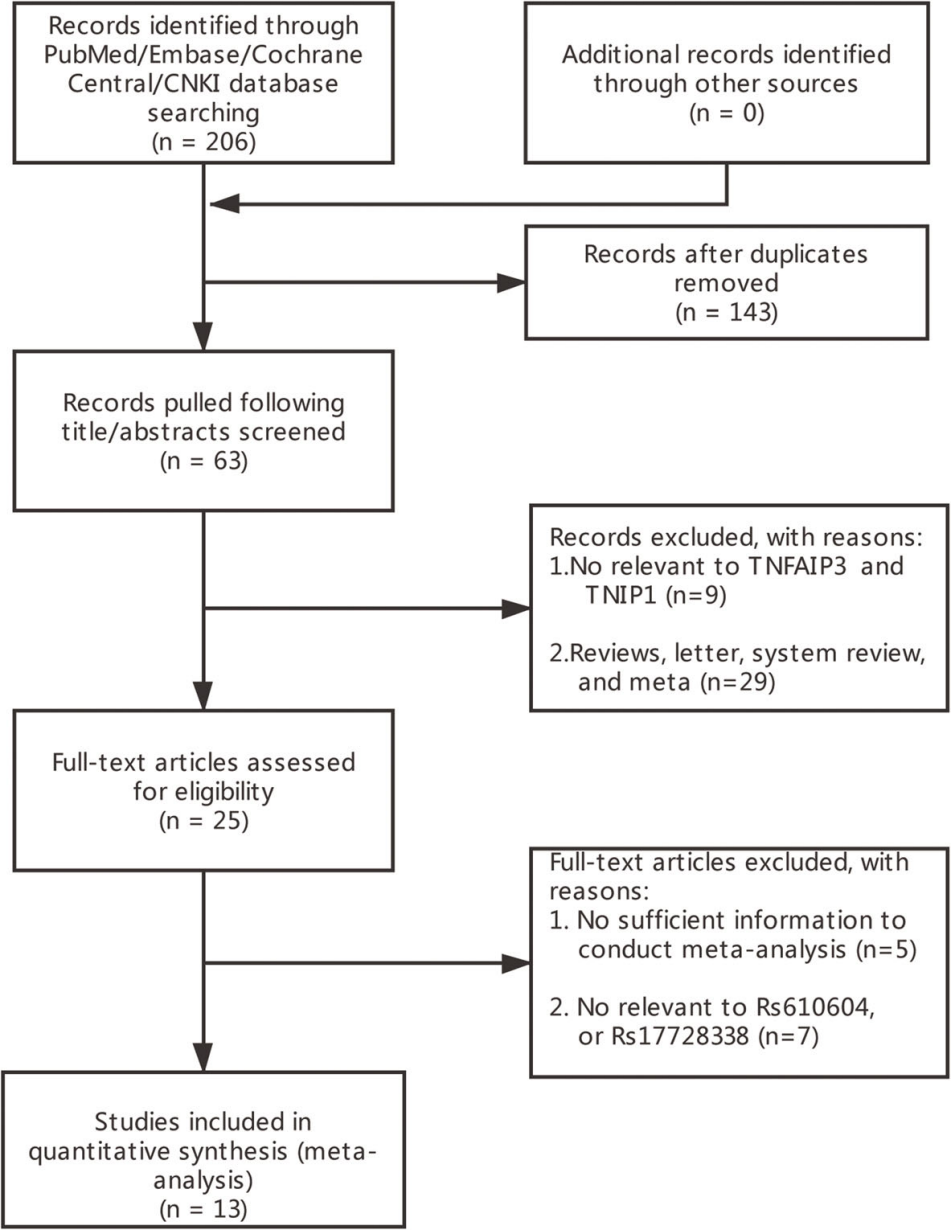
Flow diagram of literature search and screen

Initial retrieval of the four databases harvested 206 records: 43 from China National Knowledge Infrastructure (CNKI), 72 from PubMed, 80 from Embase, and 11 from Cochrane library. After removed duplicated and irrelevant records. 13 articles ultimately went into the process of meta-analysis. The detailed process of the literature search and screen is shown in Fig. 2. Of the 13 articles, 11 articles containing original data for rs610604. These studies were performed in UK [19], Egypt [20], India [21], China [22–25], Pakistani [26], USA [27], Sweden [28], México [29]. In all, these studies included 11,556 psoriasis patients and 16,720 controls. As for rs17728338, there were 9 articles involved in them, and they were conducted in UK [19], Europe [30], China [22, 23, 31], India [21], Pakistani [26], USA [27], México [29]. In all, these studies containing 11,776 psoriasis patients and 17,631 controls. The detailed characteristics of every study for rs610604 and rs17728338 are shown in Tables 1 and 2, respectively. The results of methodological quality evaluation for each study by using NOS is illustrated shown in Table 3.

**Table 1:** Main characteristics of included studies

Study	Year	Country	Ethnicity	Case/Control	Case		Control		HWE
					M	W	M	W	
*Rs610604 (T/G)*									
Bowes (1) [19]	2011	UK	UK	742/5198	520	844	3286	7110	0.04
Bowes (2) [19]	2011	UK	Ireland	161/334	102	220	235	433	1
Haase [20]	2015	Egypt	Egyptian	132/175	123	141	118	232	0.016
Indhumathi [21]	2015	India	Indian	360/360	260	460	202	518	0.223
Li [22]	2014	China	Chinese	201/300	20	382	58	542	0.754
Munir [26]	2015	Pakistani	Asian	533/373	354	712	320	746	> 0.05
Nair (1) [27]	2009	USA	Caucasian	1359/1400	1017	1701	890	1510	NA
Nair (2) [27]	2009	USA	Caucasian	5048/5051	3635	6461	3233	6869	NA
Nikamo [28]	2015	Sweden	Caucasian	1411/1529	1040	1782	979	2079	NA
Villarreal-Martínez [29]	2016	México	México	46/103	38	54	91	115	1
Yang [23]	2013	China	Chinese	974/1181	187	1761	189	2173	0.05
Zhang, C [25]	2015	China	Chinese	351/296	105	597	61	531	0.928
Zhang, Z [24]	2015	China	Chinese	238/420	62	414	84	756	0.914

*M* Mutant Allele, *W* Wild Allele, *HWE* Hardy-Weinberg Equilibrium, *NA* Not Available

**Table 2:** Main characteristics of included studies

Study	Year	Country	Ethnicity	Case/Control	Case		Control		HWE
					M	W	M	W	
*Rs17728338 (G/A)*									
Bowes (1) [19]	2011	UK	UK	742/5198	114	1370	572	9824	0.42
Bowes (2) [19]	2011	UK	Ireland	161/334	29	293	33	635	0.04
Das [30]	2015	Europe	European	2212/2855	398	4026	286	5424	NA
Han [31]	2016	China	Han population	140/476	54	226	85	867	> 0.05
Indhumathi [21]	2015	India	Indian	360/360	202	518	147	573	0.747
Li [22]	2014	China	Chinese	201/300	56	544	40	560	0.754
Munir [26]	2015	Pakistani	Asian	533/373	142	924	67	679	> 0,05
Nair (1) [27]	2009	USA	Caucasian	1359/1400	253	2465	157	2643	NA
Nair (2) [27]	2009	USA	Caucasian	5048/5051	878	9218	546	9556	NA
Villarreal-Martínez [29]	2016	México	México	46/103	11	81	11	195	0.245
Yang [23]	2013	China	Chinese	974/1181	276	1672	213	2149	1

*M* mutant allele, *W* wild allele, *HWE* Hardy-Weinberg Equilibrium

**Table 3:** Quality assessment of included studies according to the Newcastle-Ottawa Scale

Item/Study	Adequate definition of cases	Representativeness of cases	Selection of control subjects	Definition of control subjects	Control for important factor or additional factor	Exposure assessment	Same method of ascertainment for all subjects	Non-response rate	Total score
Bowes [19] 2011	1	0	1	1	1	1	1	1	7
Das [30] 2015	1	0	1	1	1	1	1	1	7
Haase [20] 2014	1	0	1	1	1	1	1	1	7
Han [31] 2016	1	0	1	1	1	1	1	1	7
Indhumathi [21] 2015	1	0	1	1	1	1	1	1	7
Li [22] 2014	1	0	0	1	1	1	1	1	6
Munir [26] 2015	1	0	1	1	1	1	1	1	7
Nair [27] 2009	1	0	1	1	1	1	1	1	7
Nikamo [28] 2015	1	0	0	1	1	1	1	1	6
Villarreal-Martínez [29] 2016	1	0	1	1	1	1	1	1	7
Yang [23] 2011	1	0	1	1	1	1	1	1	7
Zhang, Z [24] 2015	1	0	1	1	1	1	1	1	7
Zhang, C [25] 2015	1	0	0	1	1	1	1	1	6

### Meta-analyses results

#### Rs610604 polymorphism and psoriasis susceptibility

**Fig. 3:**
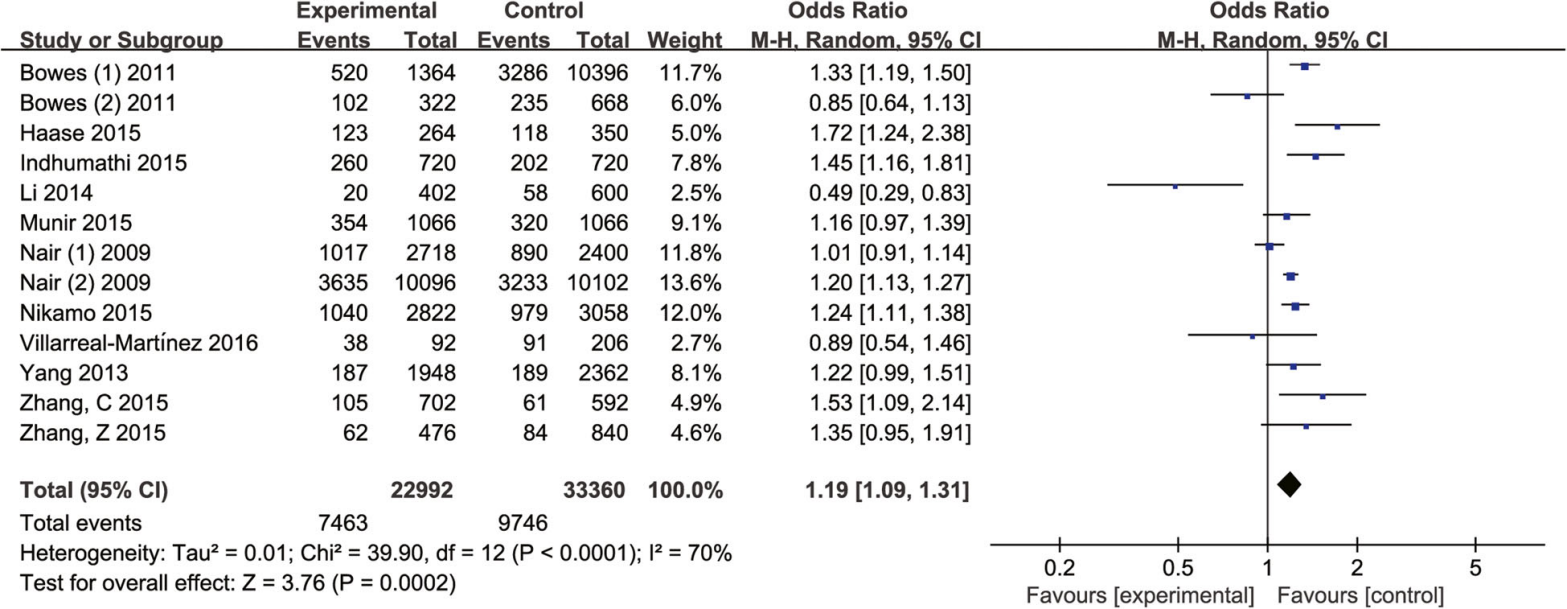
Forest plot of rs610604 in TNFAIP3 gene and risk of psoriasis

We used random-effect model to evaluate the association between rs610604 polymorphism and psoriasis vulnerability owning to a significant heterogeneity amidst all the included studies (*P* < 0.0001, *I*^2^ = 70%). The results demonstrated that rs610604 was significantly associated with psoriasis risk (G vs. T, OR = 1.19, 95% CI: 1.09–1.31, *P* = 0.0002; Fig. 3).

#### Rs17728338 polymorphism and psoriasis susceptibility

**Fig. 4:**
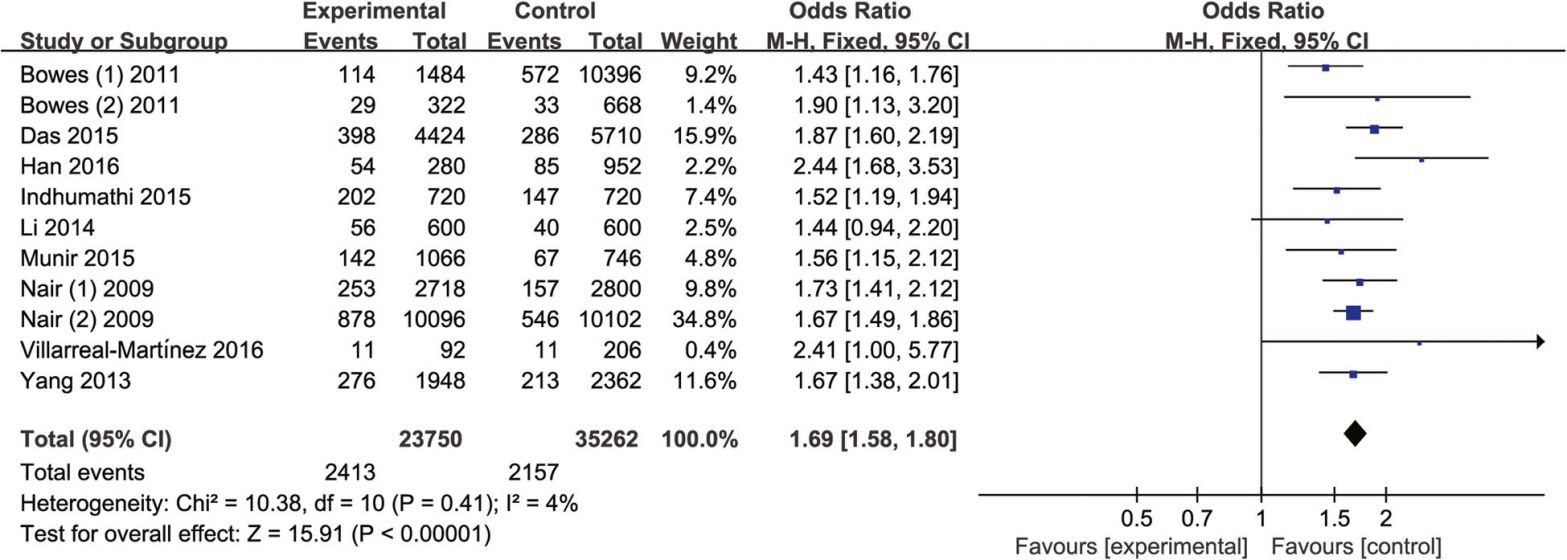
Forest plot of rs17728338 in TNIP1 gene and risk of psoriasis

Heterogeneity was small among all the studies for rs17728338 (*P* = 0.41, I^2^ = 4%). therefore, the fixed-effect model was used to conduct the meta-analysis. Our results demonstrated a significant association between rs17728338 polymorphism and psoriasis vulnerability (A vs. G, OR = 1.69, 95% CI:1.58–1.80, *P* < 0.00001; Fig. 4).

### Sensitivity analysis and publication bias

**Fig. 5:**
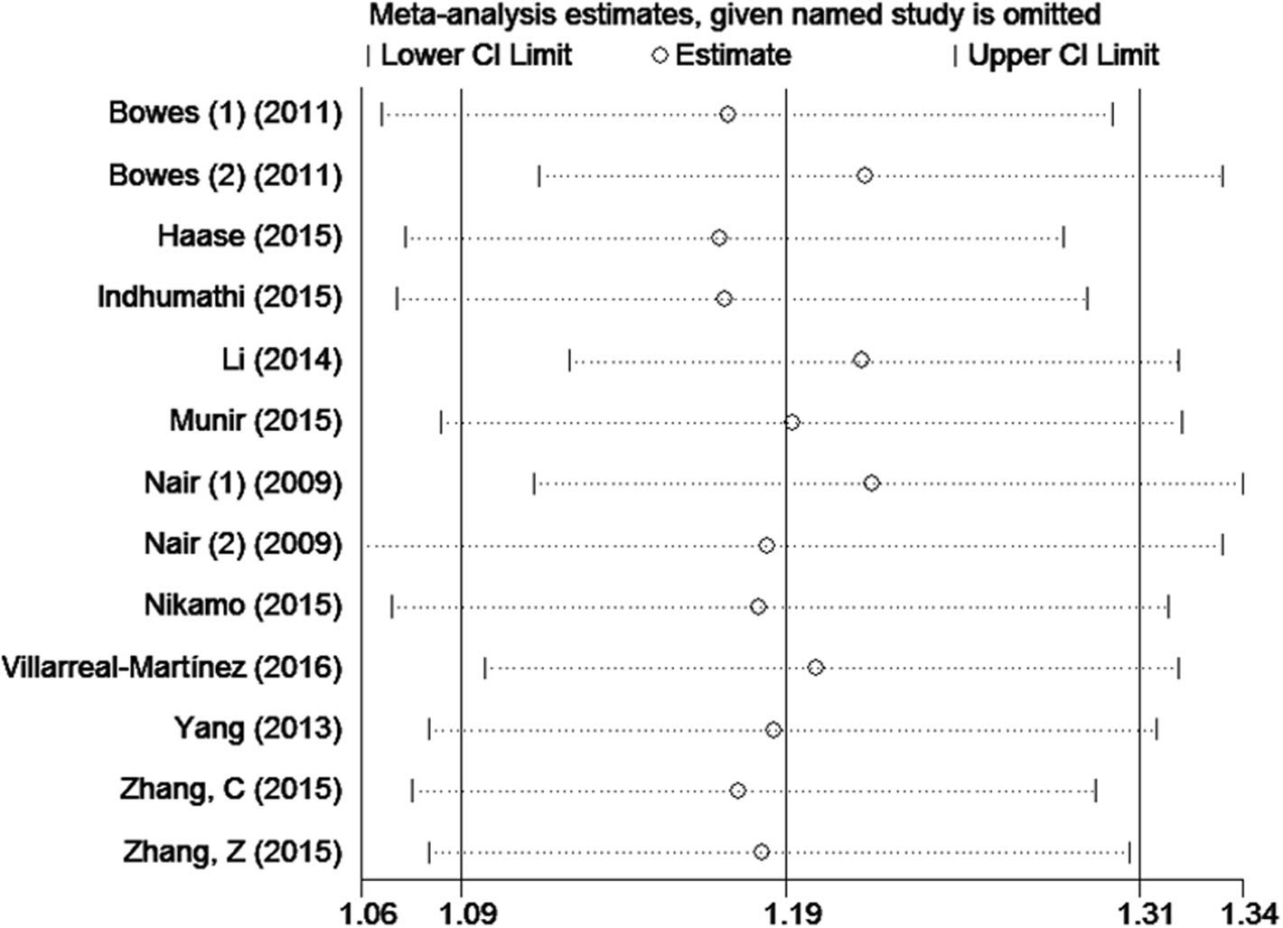
Sensitivity analysis of rs610604 in TNFAIP3 gene and risk of psoriasis

**Fig. 6:**
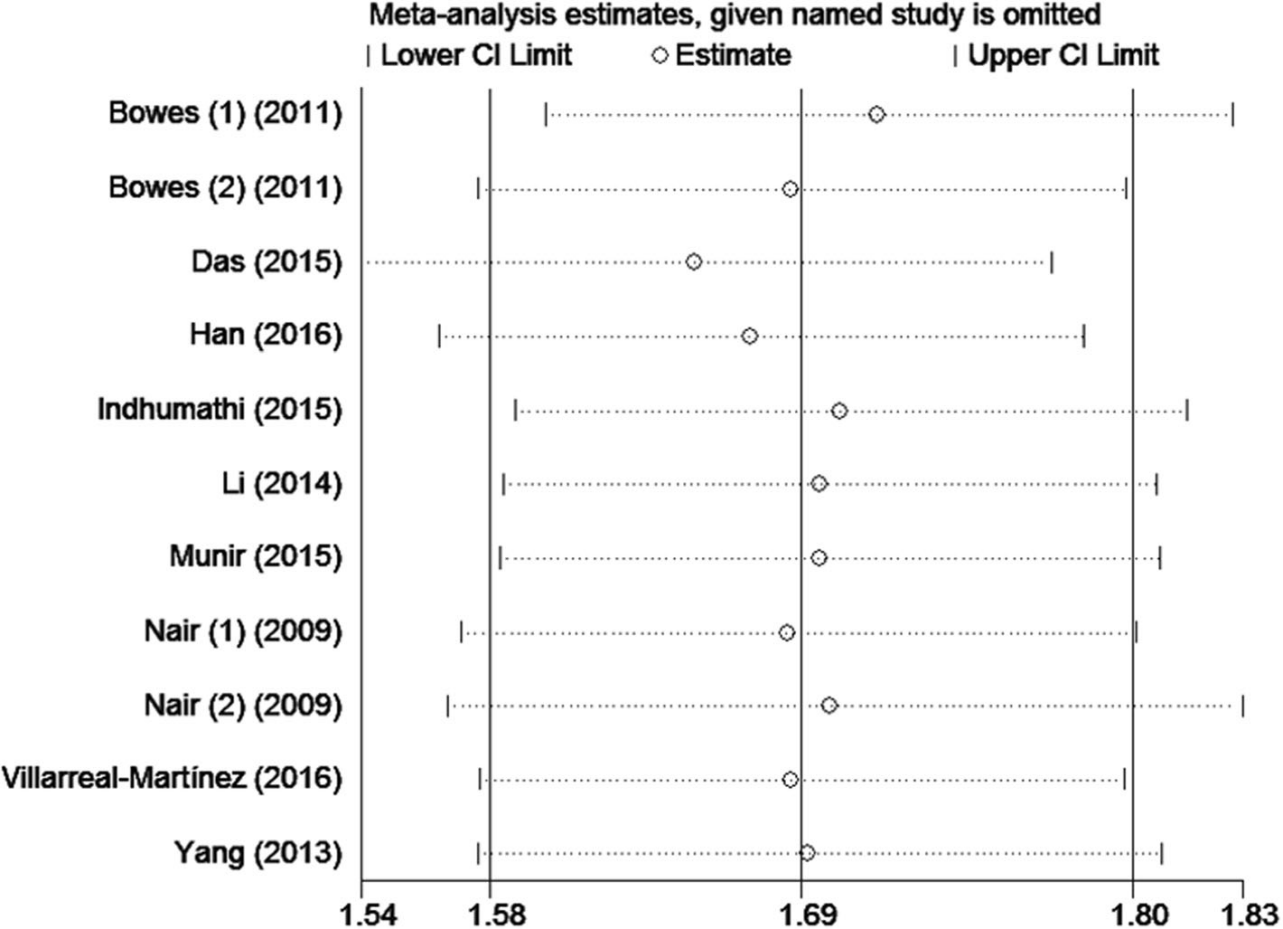
Sensitivity analysis of rs17728338 in TNIP1 gene and risk of psoriasis

**Fig. 7:**
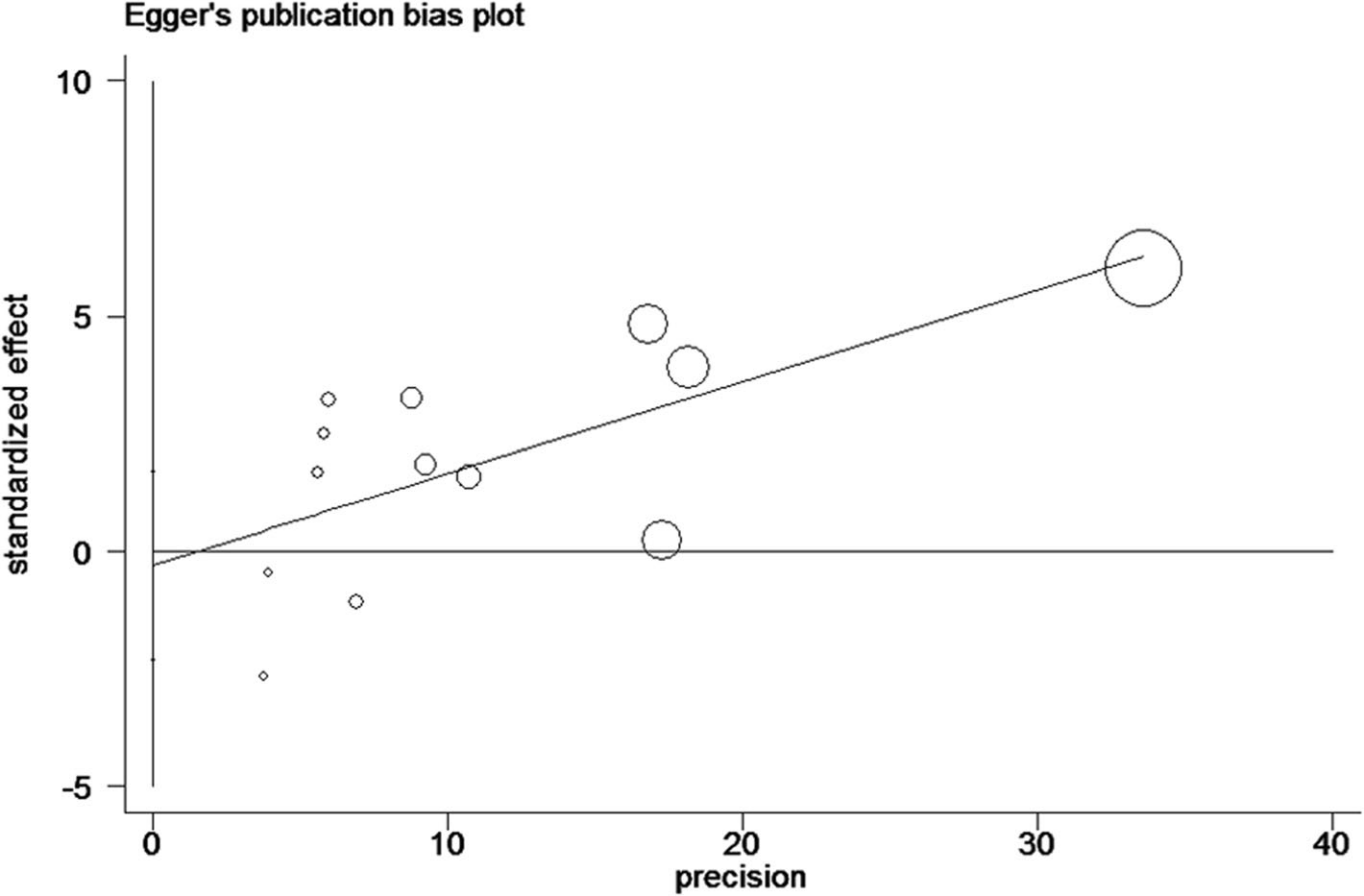
Egger’s test for rs610604 in TNFAIP3 gene and risk of psoriasis

**Fig. 8:**
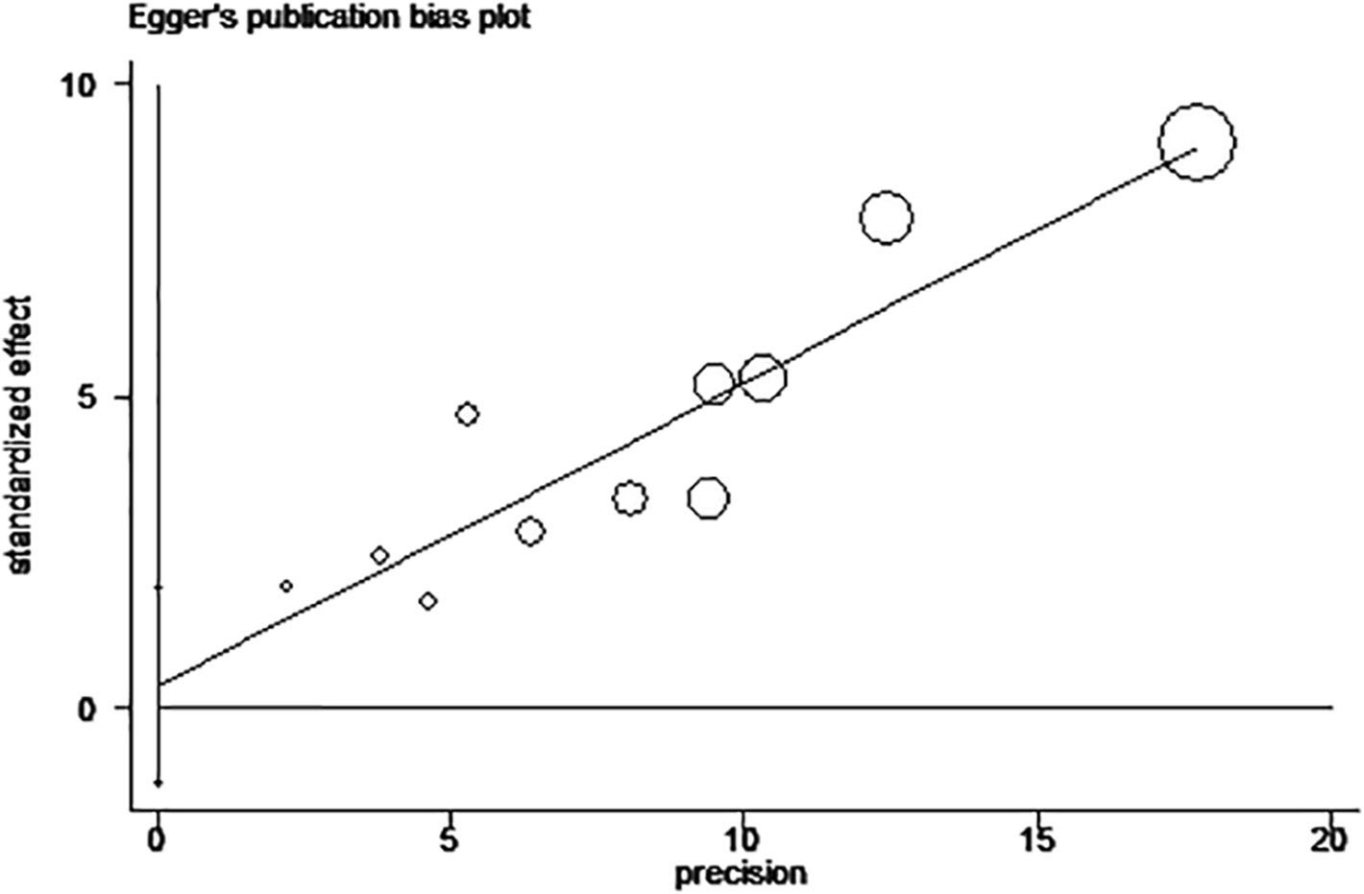
Egger’s test for rs17728338 in TNIP1 gene and risk of psoriasis

The sensitivity of rs610604 and rs17728338 analysis were visually illustrated by Figs. 5 and 6. The publication bias was shown by the Egger’s test plots of rs610604 (*P* = 0.755) and rs17728338 (*P* = 0.616) (Figs. 7 and 8), suggesting that there was no statistically significant publication bias.

### Functional analysis

**Fig. 9:**
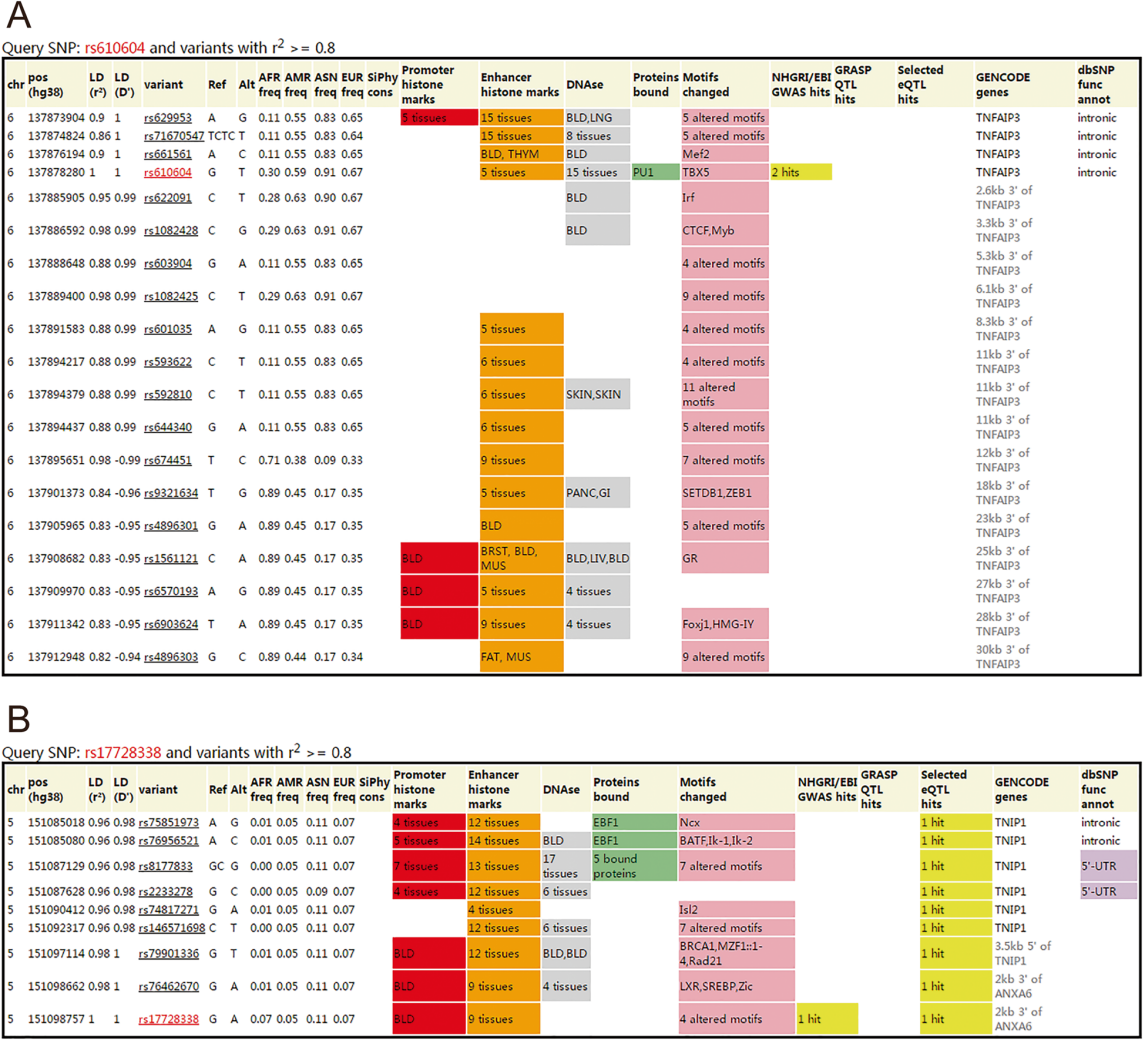
HaploReg view of rs610604 in TNFAIP3 and rs17728338 in TNIP1 gene using HaploReg version 4.1 (http://pubs.broadinstitute.org/mammals/haploreg/haploreg.php). **a** rs610604; **b** rs17728338

The functional analysis was undertaken by using HaploReg are displayed in Fig. 9.

## Discussion

Although the precise mechanism of human psoriasis remains somewhat enigmatic. It is increasingly recognized that strong genetic predisposition act as an intrinsic factor for psoriasis pathogenesis, and SNPs in the human genome may be one of the keys to unlock insights into the genetic basis for the occurrence, development and relapse of psoriasis [32, 33]. As intrinsic factors, polymorphisms in TNFAIP3 and TNIP1 gene has garnered considerable attention over the past decade years by different research teams all over the world.

Our results indicated that there is a statistically higher frequency of the rs610604 G in psoriasis patients. Most individual studies were in accordance with the result analysis by synthesis. The results of the meta-analysis remained unchanged when we conducted the leave-one-out sensitivity analysis. For rs17728338, the pooled outcome illustrated that the A allele of rs17728338 has a significantly increased risk for psoriasis. To further explore the underlying mechanisms of the interaction of TNFAIP3 and TNIP1, the HaploReg 4.1 online database was used to predict the functions of the two loci in silico. According to HaploReg, enhancer histone marks for rs610604 were found in 5 different human tissues, while enhancer histone marks for rs17728338 were found in 9 different human tissues. Both rs610604 and rs17728338 were in linkage disequilibrium with numerous other loci using a threshold of r^2^ ≥ 0.8. Regulatory motifs changed were found in both rs610604 and rs17728338. These in silico information may help to have a better understanding of the functions of the two loci, and functional experiments are strongly needed to validate these hypotheses in the future.

A20 was first characterized as a cytokine-inducible factor by a seminal study of Dixit, V. M. et al. in 1990 [34]. After that, in the year of 2004, Dixit and co-workers discovered that A20 was involved in TNF-induced NF-кB activation by playing the role of dual ubiquitin-editing enzyme [35, 36]. Dysregulation of A20 expression was found to be associated with inflammatory and autoimmune disease such as psoriasis as well as the pathogenesis of cancer over the past few years. Jiang et al.’s study [37] suggested that TNFAIP3 mRNA expression level significantly correlated with the severity and pathology of psoriasis. Other studies on systemic lupus erythematosus (SLE) and type 2 diabetes reported that some of the single nucleotide polymorphisms (SNPs) could influence the expression level of the TNFAIP3 [38, 39]. As for cancer involvement, A20 mRNA was found to be upregulated in the poorly differentiated head and neck squamous cell carcinomas (SCCs) of the skin while no A20 mRNA is observed in normal tissues samples [40]. The molecular mechanism of A20 functions underlying these biological processes is generally characterized as the inhibitory effect of NF-κB activation by editing the ubiquitylation status of its numerous proximal signaling proteins such as receptor-interacting protein serine/threonine kinase 1 (RIPK1), TNF Receptor Associated Factor 6 (TRAF6), Mucosa-Associated Lymphoid Tissue Lymphoma Translocation Protein 1 (MALT1), etc. [41, 42] Apart from NF-κB signalling pathway, A20 has also been reported to be involved in the regulation of other signalling circuits including Wnt pathway, interferon regulatory factor (IRF) pathway, etc. [43, 44] There were also studies focusing on blocking autophagy and anti-apoptotic activities by deubiquitination [45]. However, the exact mechanisms by which it does this remains unclear. More researches are needed to explore the mechanisms underlying them.

One of the most critical A20 binding protein is TNFAIP3 Interacting Protein 1 (TNIP1), which has another alias of ABIN-1. It has been reported that TNFAIP3 and TNIP1 physically interact with each other to inhibit cell death and NF-κB signalling pathway [46, 47]. Similar with TNFAIP3, more than 3 genome-wide association studies (GWAS) indicated that TNIP1 had been implicated in numerous inflammatory disease, including psoriasis, psoriatic arthritis, systemic lupus erythematosus (SLE), systemic sclerosis (SSC), rheumatoid arthritis (RA) [19, 27, 48–50]. It is probably that A20 collaborate with TNIP1 to be involved in the pathophysiology of these diseases.

To date, this is the first comprehensive study on the correlation between TNFAIP3 and TNIP1 polymorphisms and psoriasis vulnerability. However, several drawbacks should not be overlooked. First, as far as a limited number of studies are concerned, although we have collected all the currently related data, false negatives of our study may exist. Second, the genetic factor for psoriasis is composed of multiple genes and loci. However, we only concentrated on rs610604 in TNIP1 and rs17328338 in TNFAIP3 in this work. Third, only the allele model was used to analyze the data. Other genetic models are strongly recommended to be used as long as there are enough future relevant researches. Fourth, the HWE of some included studies were missing, which may lead to Information bias. Finally, only studies published in English were finally included in the present study, which may lead to selection bias.

## Conclusion

The results of our meta-analysis suggested that G allele of rs610604 polymorphisms in TNFAIP3 and A allele of rs17728338 polymorphisms in TNIP1 were considered to have an increased risk for psoriasis.

## Data Availability

All data generated or analyzed in this study are included in this paper.

## Abbreviations

SNP: Single nucleotide polymorphism
CNKI: China National Knowledge Infrastructure
NOS: Newcastle–Ottawa scale
TNFAIP3: Tumor Necrosis Factor Alpha-Induced Protein 3
TNIP1: TNFAIP3 Interacting Protein 1
OR: Odds ratio
CI: Confidence interval
HWE: Hardy-Weinberg Equilibrium
GWAS: Genome-wide association study
RIPK1: Receptor-interacting protein serine/threonine kinase 1
TRAF6: TNF Receptor Associated Factor 6
MALT1: Mucosa-Associated Lymphoid Tissue Lymphoma Translocation Protein 1
IRF: Interferon regulatory factor
SLE: Systemic lupus erythematosus
SSC: Systemic sclerosis
RA: Rheumatoid arthritis
